# Huntington’s disease: a forensic risk factor in women

**DOI:** 10.1186/s40734-019-0078-x

**Published:** 2019-07-24

**Authors:** Elvina May-Yin Chu, Mari O’Neill, Debasish Das Purkayastha, Caroline Knight

**Affiliations:** 1 Huntington’s Disease Centre, UCL Institute of Neurology, 2nd Floor Russell Square House, London, WC1B 5EH UK; 2 Department of Psychiatry, Queen’s University, Kingston, ON Canada; 3 Clinical Psychology, Priory Group, Melton Mowbray, UK; 4 General Adult Psychiatry, Northamptonshire Healthcare Foundation Trust, Northampton, UK; 5 Elysium Neurological Services, Elysium Healthcare, St Neots Hospital, St Neots, Cambridgeshire UK; 6 The Oakleaf Group, Hartwell, Northamptonshire UK; 7 School of Psychology, University of Leicester, Leicester, Leicestershire UK

**Keywords:** Huntington’s disease, Neuropsychiatry, Forensic risk

## Abstract

**Background:**

Huntington’s disease (HD) is an autosomal dominant, neurodegenerative disorder. Associated cognitive deficits including impulsivity and disinhibition are the same factors that also predispose to forensic risk. Men tend to be perpetrators of more severe violent behaviours than women and women are less likely than men to be arrested for violence. This finding is not applicable in the case of women with Huntington’s disease and explored in the three clinical cases of women with HD and their forensic histories that are subsequently described.

**Case presentation:**

‘A’ was admitted from court following a charge of arson and reckless behavior, with increasing severity and frequency of self-harm and attempted suicide. This case demonstrates someone who had previously presented to psychiatric services on multiple occasions for various reasons, culminating in a serious criminal charge of arson due to psychiatric symptoms associated with HD.

‘B’ was arrested and imprisoned after having been charged with actual bodily harm (ABH) for assaulting her partner and young daughter then breaking her bail conditions. Although she was gene positive for HD she had no neurological symptoms of the disease. *B* was given leave but needed to be recalled to hospital by police. Six weeks later the medical recommendation for a court imposed hospital order was overturned as *B* presented and articulated her case so convincingly in court. This case demonstrates that even in the absence of psychiatric history or movement disorder there may be substantial forensic risk indicated by subtle underlying cognitive deficits due to changes in executive function affecting the frontal lobes.

‘C’ was admitted to acute psychiatric services after being found wandering in traffic wanting to die. She had been diagnosed with HD in the previous year and had a long criminal record on a background of alcohol dependency. Following transfer to a specialist psychiatric unit, she engaged well with a neurobehavioural levels system which rewards desirable and appropriate behaviours and she responded well to a highly structured environment resulting in discharge to a community placement.

**Conclusions:**

These three case studies aim to highlight the need to raise awareness of the increased forensic risk in women with HD. Although criminal behaviour is less frequently observed in women than men and usually violence is less severe in women, HD may cause or contribute to criminal behaviour that can be violent in nature in women who are gene carriers for HD even in the absence of movement disorder, psychiatric symptoms or overt cognitive deficits. Assessment and earlier treatment in appropriate hospital settings may successfully contain and modify behaviours leading to reduced levels of risk and recidivism in this vulnerable patient group.

## Background

Huntington’s disease (HD) is a neurodegenerative disease characterized by motor, cognitive, and psychiatric abnormalities [1]. Subcortical neurodegeneration results in significant frontal lobe deficits due to disruption specifically between the striatum and frontal subcortical circuits. This explains the frequently observed psychiatric and behavioral disturbances of apathy and executive dysfunction; including disinhibition, disorganisation and poor judgement which can be observed long before motor symptom onset [1, 2].

A triplet expansion on the Huntingtin gene (HTT) gene is responsible for production of mutant Htt [3]. Build up of toxic mutant Htt results in neurodegeneration initially affecting the striatum, leading to mental inflexibility, disinhibition, irritability and aggression [4]; which are features also relevant to criminal behaviour. A Danish study found that crime rates were significantly increased when HD gene carriers were compared to healthy first degree relatives and controls, in particular for drink driving in men but not women with HD [5].

In one HD study; apathy, disinhibition, and executive dysfunction ratings were found to be related to neurological symptom severity. Self-ratings by patients with less severe motor symptoms were comparable to informant ratings, suggesting an intact awareness of deficits; while self-ratings by patients with more severe motor symptoms were discrepant from informant data, with informants providing more severe ratings than patients [6]. This finding suggests that patients loose insight into their behavioural problems as motor symptoms progress.

Research indicates that women inflict less serious injuries and are less often arrested for violence than men [7]. A study designed explicitly to explore differences between men and women in high security psychiatric hospital using the HCR-20 (a tool to measure risk of violence by probing history, clinical presentation and risk) found no differences between men and women on HCR-20 subscales or total scores [8]. Women demonstrated lower scores for “Prior Violence” (suggesting less serious violence), “Young Age at First Violent Incident,” “Substance Abuse,” and “Negative Attitudes” but higher scores on “Personality Disorder”, “Impulsivity,” and “Stress” items. The paper confirmed earlier research suggesting mentally disordered women display similar risk factors to mentally disordered men, but concluded their patterns of violence differ. Women are typically involved in less serious forms of violence directed toward either themselves or staff in an institutional context, while men tend to perpetrate more severe forms of violence in community settings.

## Case presentations

The following case series describes three women who were gene carriers for HD. Although each patient had genetic testing for HD confirming a CAG triplet repeat expansion, the precise repeat length was unavailable. Due to the severity of their criminal behaviours and forensic risk, they were admitted to a neuropsychiatry unit in a psychiatric in-patient hospital setting. These cases highlight the need for awareness of increased risk of criminal behaviours in women who are gene positive for HD and how this neurodegenerative condition may contribute or predispose to criminal behaviour.

### Case A

*A* was a 50 year old, admitted to psychiatric hospital under Section 38 of the UK Mental Health Act (which allows a court of law to send a patient to hospital for assessment and treatment before being sentenced) after she pleaded guilty to a charge of arson. She was brought to court by her landlord for deliberately setting fire to her home. At time of transfer to neuropsychiatry, she had neurological features typical of early stage HD including chorea and dystonia.

*A* was the main carer for her mother who had HD. Aged 27 yrs. she developed depression and anxiety becoming morbidly obese as she would comfort eat when anxious. A positive genetic test for HD at 43 yrs. triggered the first of many suicide attempts, suicide threats and episodes of self-harm. Several years later, events culminated with neighbours calling emergency services to put out a fire in her living room and she was arrested on suspicion of arson.

On admission *A* stated she would rather die than live with HD and was placed on close supervision as fire setting remained a risk. Increased physiotherapy input resulted in improved gait and posture through exercise and she enjoyed escorted community leave. A behavioural program using a token economy to address her aggression was implemented and home visits subsequently facilitated. Two years after admission there was an increase in levels of anger and frustration which the patient herself attributed to worsening mobility and falls. Cognitive decline with marked impulsivity was evident and she frequently required seclusion. Three years later she was discharged to a nursing home as she was no longer independently mobile and risk to self and others was therefore much reduced.

### Case B

*B* was a 45 yr. old woman with premanifest HD admitted to psychiatric hospital under Section 38 (an hospital order). This was her first contact with psychiatric services. Prior to admission to neuropsychiatry B was charged with Actual Bodily Harm after she assaulted her partner and their young child. She was released on bail but subsequently placed on remand (detained in prison while awaiting trial after being arrested and charged for a criminal offence) after breaking bail conditions by breaking into and entering the family home despite a police restriction order. She had no movement disorder symptoms at this time and no psychiatric history. Although there was known HD in the paternal family line, her father died from complications of Crohn’s disease before developing any signs of HD.

B had been living with her partner and they had three children together. She graduated and worked in nursing before taking a postgraduate degree. She then renovated properties with her partner and also continued nursing part-time. Problems began several years earlier when she flew into a fit of rage overturning the dinner table after her partner undercooked the meat for Christmas dinner. There followed multiple episodes of domestic violence; she repeatedly hit her partner on the head with a mirror and radio in anger, she broke windows at home and had allowed an unpaid parking fine to escalate. On one occasion she slapped her frail and elderly grandmother. Police were called to the family home numerous times but charges were never pressed by her partner.

Throughout admission, *B* remained well presented, articulate with unremarkable mental state examination. Although full scale IQ (FSIQ) 89 was within normal range, estimated premorbid IQ 105 indicated cognitive decline, with a marked discrepancy between verbal and performance IQ (VIQ = 97, PIQ = 79). She demonstrated chaotic planning in the zoo map task assessing frontal executive abilities stating she had, “No choice but to break the rules”. Kitchen assessment with the occupational therapist found that she was unable to estimate time required for tasks and she could not budget for ingredients. Although there were no pathognomonic motor signs of HD, she was markedly slow at the 9 Hole Peg Test (right = 21.5 s, left = 32 s).

*B* was given leave to her sister’s home but problems occurred when she lost her youngest daughter in a crowded shopping area. She returned alone to her sister’s house instead of searching for her child, this caused a family argument and she stormed out of the house, needing to be recalled to hospital by police. Six weeks later, the recommendation for a section 37 of the MHA (allowing the court to send someone to hospital instead of prison) was overturned and *B* self-discharged prematurely before any community support could be put in place for her.

### Case C

*C* was a 42 yr. old woman admitted to hospital after police found her walking in traffic with suicidal intent. At the time, neurological features were typical of early stage HD with mild chorea and a broad based gait.

*C* described a strict Catholic up-bringing and an unhappy childhood in an impoverished Italian immigrant family. There was no known history of Huntington’s disease in the family but both parents had passed away prematurely from cancer. As a child C was raped by her brother and described subsequently “never feeling good enough” despite achieving a university education. Psychiatric history was of alcohol dependency leading to downward social drift and eventual homelessness, this was exacerbated by difficulties coming to terms with a positive HD gene test. Her criminal record included: alcohol related driving offences, shoplifting, breech of conditional discharge, failure to comply with community order requirements, skipping bail, drunk on highway in a public place, drunk and disorderly and assaulting police. Although rehoused in a local authority flat, this was occupied by strangers who abused and exploited her in the community and she had attended hospital for alcohol detoxification and treatment of domestic violence injuries.

On admission *C* appeared dishevelled, irritable and suspicious with choreiform movements. She reported low self-esteem, guilt feelings and a death-wish. Neuropsychological assessment confirmed FSIQ = 70 (borderline), with a discrepancy between verbal and performance IQ (VIQ = 78, PIQ = 66), with extremely poor visuo-spatial abilities, language, attention, delayed memory, and impaired frontal function with difficulty planning and problem solving. Poor balance was noted (Berg scale 34/56) and she had several falls on the ward. Due to chorea, she would often accidentally drop cigarettes while smoking and was therefore at risk of burns and a fire hazard. Kitchen assessment highlighted safety concerns due to inability sequencing preparation of components for a meal and poor time monitoring. Personal hygiene required prompting and she was easily distractible when crossing roads.

She engaged well with a neurobehavioural levels system which rewards desirable and appropriate behaviours and she responded well to a highly structured ward environment resulting in successful discharge to a community placement 2 years later.

## Discussion and conclusions

Suicidal ideation is common in patients with HD [9] and parasuicide or psychiatric hospital admissions can occur, especially following the stress of predictive testing [10]. Over 25% of HD patients acknowledge at least one suicide attempt, especially when significant functional decline is evident [11]. Increased suicide risk is also associated with depression and symptoms of major depression can precede the movement disorder in HD by many years [12]. Repeated episodes of self-harm and parasuicide as seen in case A are also typical behaviours of borderline personality disorder, HD can exacerbate such borderline traits and psychiatric evaluation is therefore necessary to differentiate between depression and borderline personality.

Aggressive behaviour can be observed, even in premanifest HD when no motor symptoms are observed as described in case B. Dysfunction in the prefrontal cortex brain circuits that normally inhibit emotional impulses is a prelude to violent outbursts [9]. A lack of social problem solving skills may be reflective of cognitive deficits rather than psychiatric mental state [13] with irritability giving rise to outbursts of aggression and violence due to inability to generate any further solutions to problems faced [14].

Alcohol is a CNS depressant, intoxication contributes to disinhibition and impulsivity with the HD brain being more vulnerable to effects of alcohol [15]. Recidivism may be compounded by increased severity and frequency of criminal behaviour as reflected in case C due to increasing vulnerability to effects of alcohol as a result of progressive neurodegeneration in HD.

Women with serious medical and psychological disorders and their caregivers benefit significantly from social problem solving-based interventions, resulting in decreased depression, decreased impulsive/aggressive behavior, and improved social functioning and quality of life [16]. HD patients in early stage disease, may demonstrate improved mental health and cognitive function following such interventions [13]. This supports the notion that earlier assessment and admission to specialist psychiatric services for behavioural management may limit forensic risk.

Factor analysis has indicated that irritability is related to impulsivity and aggression [17] and low serotonin levels are linked to criminal behavior [18]. Impulsive rather than carefully planned crimes are especially likely to be committed by those exhibiting serotonergic malfunctioning with affected areas corresponding to serotoninergic pathways connecting the prefrontal and limbic system areas of emotion [19]. In premanifest HD before onset of any motor symptoms, task induced irritation results in a different pattern of neuronal activation compared to healthy controls as seen on fMRI in areas involving the amygdala and orbitofrontal circuits. This attenuated modulation of emotional status increases the risk for developing symptoms such as irritability [14]. The key to management of irritability and aggression is to place the behaviour in context by identifying and avoiding precipitants [11] in the cases described here, these included falls, presence of young children and police.

The Evolutionary Neuroandrogenic Theory postulates that sex hormones alter male brain functioning in ways that promote competitive/victimizing behaviour, which includes criminal behaviour directed at others [20]. It also implies that serotonin facilitates executive cognitive functioning required to restrain impulsive behaviour, especially impulsive responses to rage and persistent frustration. Lesions to orbito-frontal circuits affected by striatal degeneration typically result in irritability and impulsivity but manipulation of the serotonergic system with selective serotonin receptor inhibitors (SSRIs) may reduce irritable or aggressive behaviours [21] suggesting this may be a beneficial medication in those with HD both in treating irritability that may lead to aggression and also depression.

Successful treatment of refractory depression in HD using deep transcranial magnetic stimulation (dTMS) has been reported as a case study [22], while repetetive transcranial magnetic stimulation (rTMS) has also been reported to decrease severity of HD motor symptoms when the motor cortex is stimulated [23] and is a non-pharmacological treatment with minimal side effects.

A pharmacological review in HD found a lack of RCTs in the published literature but found that second generation antipsychotic medications are typically useful in treatment of chorea when psychiatric symptoms were also present and preferable due to a more limited EPSE profile [24]. Authors (EC, DP) have personal experience of treating severe aggression in HD with olanzapine and the side effects of sedation and appetite stimulation causing weight gain are often favourable in HD.

Monoamine oxidase (MAO) is an enzyme found throughout the body. In the brain, MAO helps to break down and clear away neurotransmitters such as serotonin, part of which often lingers in the synaptic gap after activating adjacent nerve cells [25]. Studies indicate that low platelet MAO activity is associated with increased offending rates [26] and offence-related behaviours including impulsivity and general aggression appear related to MAO polymorphisms [27]. Altered MAO activity has been observed in HD and MAO inhibitors in an HD mouse model restored dopamine, serotonin and norepinephrine levels in the striatum, reducing anxiety and depression [28]. MAO inhibitors may therefore have a role in clinical treatment of HD.

Evolutionary biology has determined that males are generally more aggressive than females in most animal species. This is due to physiological differences such as physical build, the reproductive role of females and biological cost; aggression and violence resulting in death of a male does not impact offspring in the same way as maternal death [29]. In humans, men worldwide engage in aggressive and acquisitive crimes (especially those involving violence) to a greater extent than women [30]. General Strain Theory (GST) postulates that men are more likely to commit crimes than women due to a different emotional response to strain and are more likely to respond to anger or strain with crime [31]. Evolutionary psychologist Anne Campbell [32] concludes that differences in aggressive behaviour reflect gender differences in the strength of factors controlling the behavioural expression of anger; which is why developmental studies show girls generally score higher on empathy measures, are more fearful and are better at controlling their behaviour. It would appear from the cases described that HD can result in a significant loss of control in behavioural expression of anger in women.

HD gene carriers have been reported to have a higher prevalence of criminal behavior vs. their non-carrier family members [5]. Estimates for criminal behaviour in HD are between 18 and 27% [33]. Other neurodegenerative disorders that also predispose to higher rates of violence and aggression are fronto-temporal dementia (40%) and senile dementia (27%).

In conclusion, women who are HD gene carriers may be at increased risk of criminal behaviours and a forensic history even before onset of any motor symptoms. Early assessment and treatment in appropriate hospital settings may contain and modify behaviours leading to reduced risk and recidivism.

## Data Availability

Not applicable.

## Abbreviations

CAG: Cytosine, arginine, guanine
FSIQ: Full-scale intelligence quotient
GST: General strain theory
HD: Huntington’s disease
HTT: Huntingtin gene
Htt: Huntingtin protein
PIQ: Performance intelligence quotient
SSRIs: Selective serotonin reuptake inhibitors
VIQ: Verbal intelligence quotient

## References

[1] Paulsen J (2001). Neuropsychiatric aspects of Huntington’s disease. J Neurol Neurosurg Psychiatry.

[2] Thompson J, Snowden J, Craufurd D, Neary D (2002). Behavior in Huntington’s disease: dissociating cognition-based and mood-based changes. J Neuropsychiatry Clin Neurosci.

[3] Chu E (2014). Huntington’s disease: from basic science to clinical treatment. Neuropsychiatry News.

[4] Craufurd D, Snowden J (2014). Neuropsychiatry and Neuropsychology. In: Bates G, Tabrizi S, editors. Huntington’s Disease.

[5] Jensen P, Fenger K, Bolwig T, Sorensen S (1998). Crime in Huntington’s disease: a study of registered offences among patients, relatives and controls. J Neurol Neurosurg Psychiatry.

[6] Hergert D, Blinkoff D (2015). Examining Huntington’s disease patient and informant concordance on frontally mediated behaviors. J Clin Exp Neuropsychol.

[7] Monahan J, Steadman H, Frost L, Bonnie R (2001). Violence risk assessment: A quarter century of research. The evolution of mental health law.

[8] Strand S (2005). Gender differences in psychopathy in a Swedish offender sample. Behav Sci Law.

[9] Hubers A, van Dujin E, Roos R (2013). Suicidal ideation in a European Huntington's disease population. J Affect Disord.

[10] Almqvist E, Bloch M, Brinkman R, Craufurd D, Hayden M (1999). A worldwide assessment of the frequency of suicide, suicide attempts, or psychiatric hospitalization after predictive testing for Huntington disease. Am J Hum Genet.

[11] Wetzel H (2011). Suicidal ideation in Huntington disease: the role of comorbidity. Psychiatry Res.

[12] Rosenblatt A (2007). Neuropsychiatry of Huntington’s disease. Dialogues Clin Neurosci.

[13] Van Liew C (2013). The functional implications of motor, cognitive, psychiatric, and social problem-solving states in Huntington’s disease. Psychiatry.

[14] Klöppel S, Stonnington C, Petrovich M (2010). Irritability in pre-clinical Huntington’s disease. Neuropsychologia.

[15] Vaughan Courtney L., Stangl Bethany L., Schwandt Melanie L., Corey Kristin M., Hendershot Christian S., Ramchandani Vijay A. (2019). The relationship between impaired control, impulsivity, and alcohol self-administration in nondependent drinkers. Experimental and Clinical Psychopharmacology.

[16] Elliott T, Berry J, Grant J (2009). Problem-solving training for family caregivers of women with disabilities: a randomized clinical trial. Behav Res Ther.

[17] Craufurd D, Thompson J, Snowden J (2001). Behavioral changes in Huntington disease. Neuropsychiatry, Neuropsychol Behav Neurol.

[18] Virkkunen M, Goldman D, Linnoila M (1996). Serotonin in alcoholic violent offenders. CIBA Found Symp.

[19] Davidson R, Putnam K, Larson C (2000). Dysfunction in the neural circuitry of emotion regulation-a possible prelude to violence. Science.

[20] Darby R (2018). Neuroimaging abnormalities in neurological patients with criminal behavior. Curr Neurol Neurosci Rep.

[21] Ranen N, Lipsey J, Treisman G, Ross C (1996). Sertraline in the treatment of severe aggressiveness. Clin Res Rep.

[22] Davis M, Philips A, Tendler A, Oberdeck A (2016). Deep rTMS for neuropsychiatric symptoms of Huntington’s disease: case report. Brain Stimul.

[23] Berardelli Alfredo, Suppa Antonio (2013). Noninvasive brain stimulation in Huntington’s disease. Handbook of Clinical Neurology.

[24] Unti E, Mazzucchi S, Palermo G, Bonucelli U, Ceravolo R (2017). Antipsychotic drugs in Huntington’s disease. Expert Rev Neurother.

[25] Westlund K, Denney RM, Kochersperger LM, Rose RM, Abell CW (1985). Distinct monoamine oxidase a and B populations in primate brain. Science.

[26] Alm P, af Klinteberg B, Humble K, Leppert J, Sörensen S, Thorell LH, Lidberg L, Oreland L (1996). Psychopathy, platelet MAO activity and criminality among former juvenile delinquents. Acta Psychiatr Scand.

[27] Manuck S, Flory J, Ferrell R, Mann J, Muldoon M (2000). A regulatory polymorphism of the monoamine oxidase-a gene may be associated with variability in aggression, impulsivity, and central nervous system serotonergic responsivity. Psychiatry Res.

[28] Garcia-Miralles M, Ooi J, Ferrari Bardile C, Liang JT, George M, Drum C, Lin RY, Hayden M, Pouladi M (2016). Treatment with the MAO-A inhibitor clorgyline elevates monoamine neurotransmitter levels and improves affective phenotypes in a mouse model of Huntington disease. Exp Neurol.

[29] Georgiev A, Klimczuk A, Traficonte D (2013). When violence pays: a cost-benefit analysis of aggressive behavior in animals and humans. Evol Psychol.

[30] Ellis L, Walsh A (2000). Criminology: a global perspective.

[31] Broidy L, Agnew R (1997). Gender and crime: a general strain theory and perspective. J Res Crime Delinq.

[32] Campbell A (2013). The evolutionary psychology of women’s aggression. Philos Trans R Soc B: Biological Sciences.

[33] Liljegren M, Naasan G, Temlett J, Perry D, Rankin K (2015). Criminal behavior in frontotemporal dementia and Alzheimer disease. JAMA Neurol.

